# Miners' Nystagmus

**Published:** 1893-06

**Authors:** 


*Miners' Nystagmus.* By SIMEON SNELL, F.R.C.S.Ed. Pp. xi., 143. Bristol: JOHN WRIGHT & CO. 1892.

High praise is unquestionably due to Mr. Snell for the thorough and unprejudiced investigation he has given during the past twelve years to the subject of Miners' Nystagmus. His work, if it did not lay the foundations, has built up most of the superstructure of our knowledge of this malady. The book before us is a handsome, well-printed, and well-illustrated volume, in which Mr. Snell's inquiries and experiments are reported and his conclusions clearly stated. It will be found of interest to all engaged in coal-mining operations, as well as to doctors in charge of pitmen. Although we may not entirely concur in considering the position of the workman as of more importance than the defective illumination, we must admit that Mr. Snell's large experience and strong statements are deserving of full consideration.

